# Microwave spectroscopy of the low-filling-factor bilayer electron solid in a wide quantum well

**DOI:** 10.1038/ncomms8071

**Published:** 2015-05-07

**Authors:** A. T. Hatke, Y. Liu, L. W. Engel, M. Shayegan, L. N. Pfeiffer, K. W. West, K. W. Baldwin

**Affiliations:** 1 National High Magnetic Field Laboratory, Tallahassee, Florida 32310, USA; 2 Department of Electrical Engineering, Princeton University, Princeton, New Jersey 08544, USA

## Abstract

At the low Landau filling factor termination of the fractional quantum Hall effect series, two-dimensional electron systems exhibit an insulating phase that is understood as a form of pinned Wigner solid. Here we use microwave spectroscopy to probe the transition to the insulator for a wide quantum well sample that can support single-layer or bilayer states depending on its overall carrier density. We find that the insulator exhibits a resonance which is characteristic of a bilayer solid. The resonance also reveals a pair of transitions within the solid, which are not accessible to dc transport measurements. As density is biased deeper into the bilayer solid regime, the resonance grows in specific intensity, and the transitions within the insulator disappear. These behaviours are suggestive of a picture of the insulating phase as an emulsion of liquid and solid components.

## Abstract (web summary)

In 2D electron gases, insulating behaviour at low fractional quantum Hall filling factors is understood by the formation of an electronic Wigner solid. Here, the authors use microwave spectroscopy to evidence an electron liquid–solid mixed phase in bilayer states of GaAs/AlGaAs wide quantum wells.

## Graphical abstract

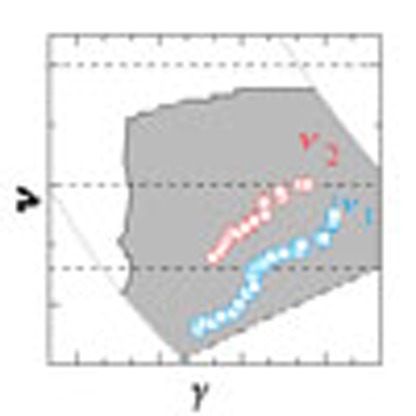

Wide quantum wells (WQWs) support bilayer as well as single-layer states, depending on the well width, *w*, and on the overall areal carrier density, *n* (refs 1, 2, 3, 4, 5, 6). A measure of the tendency of the charge in a WQW to separate into two layers is *γ*≡(*e*^2^/4*πε*_0_*εℓ*_B_)/Δ_SAS_ (refs 4, 5, 6) where *ℓ*_B_=(*ν*/2*πn*)^1/2^is the magnetic length, Δ_SAS_ is the gap between the lowest and the first-excited sub-band and *e*^2^/4*πε*_0_*εℓ*_B_ is the Coulomb energy. Figure 1a shows a phase diagram in the *γ* and filling factor, *ν*, plane. The interpretation of the insulator as a bilayer solid was inferred in ref. 5 from the phase diagram and from the response of the insulator to asymmetric gate bias that produced mismatched layer densities. At small *γ* the two-dimensional (2D) electron system (2DES) behaves like a single layer, exhibiting a one-component (1C) Wigner solid for *ν* below the 1/5 fractional quantum Hall effect (FQHE), and also for a narrow re-entrant range^7^ above it. At large *γ* the 2DES behaves as two layers with weak interaction, and the solid occurs at *ν*∼2/5, or per-layer filling *ν*_*L*_∼1/5. At intermediate *γ*, the insulator includes a *ν* range re-entrant above the 1/2 FQHE^5^,^6^, which is known to be a bilayer, interlayer-correlated state; therefore, the nearby insulator is likely a bilayer two-component (2C) solid.

Microwave spectroscopy is ideal for studies of electron solids since these exhibit pinning mode resonances^8^,^9^,^10^,^11^,^12^,^13^,^14^,^15^,^16^,^17^,^18^,^19^,^20^,^21^,^22^,^23^, in which pieces of the solid oscillate within the potential of the residual disorder. This disorder also pins the solid, rendering it insulating. The resonance peak frequency, *f*_pk_, is sensitive to solid properties, such as shear modulus and the proximity of the carriers to disorder in the host. A recent study of WQW-pinning modes^22^ focused on the small-*γ*, single-layer regime in the neighbourhood of *ν*=1. Earlier studies of bilayer pinning modes^16^,^20^,^21^ used double quantum wells, for which the layers are defined by a barrier, and 1C–2C transitions were considered in refs 20, 21.

In this paper we use microwave pinning-mode spectroscopy on a WQW to probe the low-*ν* bilayer insulator, which is understood as a bilayer electron solid. We observe two phase transitions within the solid from the pinning mode as jumps in *f*_pk_ concurrent with dips in the amplitude. We find that with increasing density the bilayer resonance is strengthened concurrent with a disappearance of the transitions. Our observations are interpreted in terms of intermediate phases, possibly involving bilayer solid and nonresonant liquid components.

## Results

### Sample details

Our measurements were performed on a GaAs/AlGaAs WQW of width *w*=80 nm with an as-cooled density of *n*=1.1 in units of 10^11^ cm^−2^, which we use throughout the paper for brevity. The microwave technique^18^,^20^,^21^,^22^ is diagrammed in Fig. 2a,b. We maintained a symmetric growth-direction charge distribution about the well centre unless otherwise noted, and calculated *γ*(*n*) from simulations (see Methods section). The sample was measured in a 60-mK bath.

### Microwave spectroscopy

The strong pinning mode shown in Fig. 3 is the evidence of a solid. We interpret the two sharp increases in *f*_pk_ as due to transitions between different solid configurations. Figure 4 gives an overview of the spectra for the *n* range we surveyed, as image plots of Re (*σ*_*xx*_) in the (*ν*, *f*) plane.

Figure 5a,b shows *σ*_pk_ (Re (*σ*_*xx*_) at the resonance maximum) and *f*_pk_ versus *ν*, respectively, for many *n*. We interpret the minima in *σ*_pk_ versus *ν* as due to the transitions, and denote the lower and higher *ν* of the transitions by *ν*_1_ and *ν*_2_, respectively. At *ν*_2_, the *f*_pk_ jump is maximal for *n*=1.26, just above the transition to the insulator from the FQHE. This jump weakens gradually as *n* increases and is absent by *n*=1.41.

The transitions within the insulator at *ν*_1_ and *ν*_2_ are most pronounced for *n* near the transition to insulator from the FQHE. Except near *ν*_1_ or *ν*_2_, the resonance *σ*_pk_ increases with *n*, as seen in Fig. 5a. At the same time, *f*_pk_ changes little with *n* except near *ν*_1_ or *ν*_2_. Pinning modes approximately obey a sum rule^15^,^24^,^25^, *πνe*^2^/2*h*=*S*/*f*_pk_, where *S* is the integrated Re (*σ*_*xx*_) versus *f* for the resonance. Figure 5c shows *S*/*f*_pk_ versus *n* for *ν*=0.4. Except near *n*=1.38, at which *S*/*f*_pk_ is suppressed, this *ν* is away from *ν*_1_ and *ν*_2_. The curve saturates as *n* goes above 1.4 to a value in reasonable agreement with the sum rule; *n*≃1.4 is approximately the largest density at which we observe the jump in *f*_pk_ at *ν*_2_, and the minima in *σ*_pk_ versus *ν* are considerably weakened by this *n*.

An important result is that the solid appears to be a bilayer. Figure 5d shows *σ*_pk_ versus *ν* for a fixed overall density of *n*=1.30, but varying the charge transfer, Δ*n*, between layers. As layer imbalance grows, *σ*_pk_ is reduced and the transition minima shift only slightly in *ν*. The interpretation is that excess carriers of the majority layer do not participate in the resonance, and reduce its strength by damping. The dc transport study in ref. 5 found the resistivity also to be sharply reduced on layer imbalance for a 75-nm WQW in this low *ν* insulator.

### Phase diagram in the γ-ν plane

**Figure 1:**
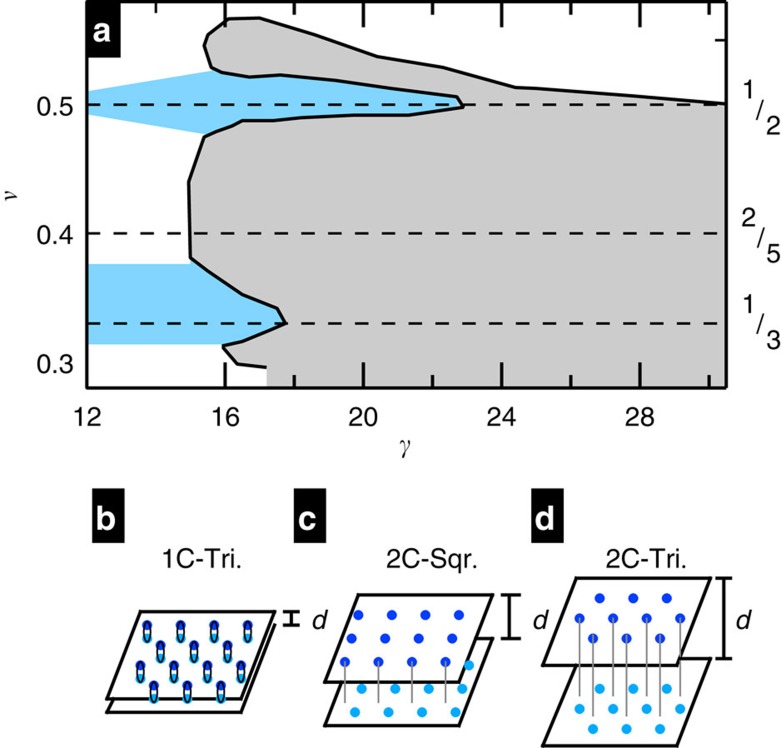
Wide quantum well overview. (**a**) Phase diagram in the *γ*–*ν* plane, where *ν* is the Landau filling and *γ* is the Coulomb energy (*e*^2^/4*πε*_0_*εℓ*_B_) divided by the interlayer tunnelling gap. The insulating area is shaded grey, and the ranges of the FQHE states at *ν*=1/2 and *ν*=1/3 are shaded blue. Horizontal dashed lines mark *ν*=1/2, 2/5 and 1/3. (**b**–**d**) Different possible structures for bilayer Wigner solids as layer separation, *d*, increases for fixed *n*. A 1C triangular (tri.) structure (**b**), proceeds to a 2C staggered square (sqr.) structure (**c**) and then to a 2C staggered triangular (tri.) structure (**d**).

Figure 6 shows the *γ*–*ν* plane with the shaded area marking the region in which a resonance is observed. Compared with the dc-transport boundary of Fig. 1a (ref. 5), the nearly vertical left boundary of the solid in Fig. 6 occurs at larger *γ*. Some of this difference is likely due to the WQW in this paper being wider, 80 nm versus 75 nm for ref. 5. It may be that relative to the dc boundary, the resonance appears further into the insulating phase. This difference between the resonance boundary and the dc boundary is what reduces the apparent depth of the rightward boundary indentation centred at *ν*=1/3. We also find no resonance in the *ν*>1/2 re-entrant insulator adjacent to the 1/2 FQHE.

## Discussion

As yet there is no theory of 2C solids in WQWs. Hartree–Fock^26^,^27^ and classical^28^ theories for double quantum wells (DQWs) admit phase transitions between different types of solids. The Hartree–Fock DQW theories^26^,^27^ predict multiple phases of bilayer solid, as sketched in Fig. 1b–d. A 1C triangular solid exists at low *n*, large interlayer separation *d* or small *γ*, while a weakly coupled, staggered triangular 2C solid exists at the opposite extreme. 2C staggered square, rhombic or rectangular lattices exist in between, when Δ_SAS_ is small enough and *d*/*l*_*B*_, which measures the ratio of intralayer and interlayer interaction energies, is in a particular (*ν*-dependent) range. The theories^26^,^27^ predict that *ν* of all transitions decreases with increasing *γ*, in contrast to the rising *ν*_1_ and *ν*_2_ versus *γ* in Fig. 6. Nonetheless, the predicted *ν*, *γ* of a solid–solid transition comes close to that of the observed transitions. For example, for *ν*∼1/3 our observed *ν*_1_ occurs for *γ*∼27.5. The lowest-*γ* transition predicted^26^ between 2C lattices (staggered square to staggered rhombic) is at *γ*∼33, for *d*/*l*_*B*_=3 and *ν*=1/3. (ref. 26 calculates up to *d*/*l*_*B*_=3 and we extrapolate a larger *γ* for *d*/*l*_*B*_∼8, which applies to the transition we see at *ν*_1_=1/3.) One source of differences between the theory and the results may be the different vertical confinements in WQWs and the DQWs for which the theory was made.

Theories^13^,^29^ of composite fermion^30^ (CF) Wigner crystals have considered single-layer systems, but predict transitions between solids of different CF vortex number 2*p*. Such transitions may explain the evolution^18^ of the pinning mode for *ν*<1/5 in lower *n* single-layer quantum well states, and may be related to the transitions of ref. 22. 2*p* is predicted to increase as *ν* decreases, where a transition from 2*p*=2–4 should occur between 1/5 and 1/6. This is in the per-layer-*ν* range of the transitions we see, but for the large *γ* case, which corresponds to two weakly coupled parallel layers, the transitions disappear. This is contrary to the expectation for the single-layer-like CF vortex-number transitions, which calculations^13^ indicate are driven by *ν*, and whose phase diagram in a single-layer quantum well was described^13^ as not sensitive to well width.

The strengthening of the resonance as *n* moves deeper into the insulator from the FQHE liquids suggests that the solid may be existing along with a component that does not produce a resonance, and that the solid fraction increases with *γ*. A series of intermediate phases with FQHE liquid and Wigner solid components was proposed theoretically^31^. The closely paired transitions at *ν*_1_ and *ν*_2_ may be between different intermediate phases. The disappearance of the transitions at approximately the *n* at which *S*/*f*_pk_ versus *n* saturates is also qualitatively suggestive of the intermediate phase picture, in which the system at large *γ* becomes a homogenous solid. Inhomogenous intermediate phases similar to those in ref. 31 can be affected by quenched disorder; the role of disorder in the presently observed phases is not clear.

In summary, microwave measurements of a WQW establish its low *ν* insulator to be a bilayer solid, since it has a pinning mode resonance that is degraded by layer-density imbalance. The resonance reveals a pair of structural transitions within the insulator. Increases in resonance strength with *γ* and the disappearance of the transitions as *S*/*f*_pk_ reaches full strength are qualitatively consistent with a picture of an inhomogeneous insulator containing resonant and nonresonant components.

## Methods

### Charge distribution in the growth direction

The density of the well was changed by front and back gates. The back gate was in direct contact with the bottom of the sample and the front gate was deposited on a piece of glass that was etched to be spaced from the sample surface to not interfere with the microwave transmission line. A symmetric, balanced, growth direction charge distribution was maintained by biasing the gates such that individually they would change the density by the same amount with equal and opposite electric fields. The asymmetric, imbalanced, distribution was obtained by first biasing one gate to get half the desired charge imbalance and then biasing the other in the opposite manner to get the same charge imbalance while maintaining the same total density with applied electric fields in equal amount and the same direction. *d* and Δ_SAS_ were calculated from simulations. The calculated Δ_SAS_ was used to obtain *γ* and had an uncertainty of about ±15% for this WQW.

### Microwave spectroscopy

**Figure 2:**
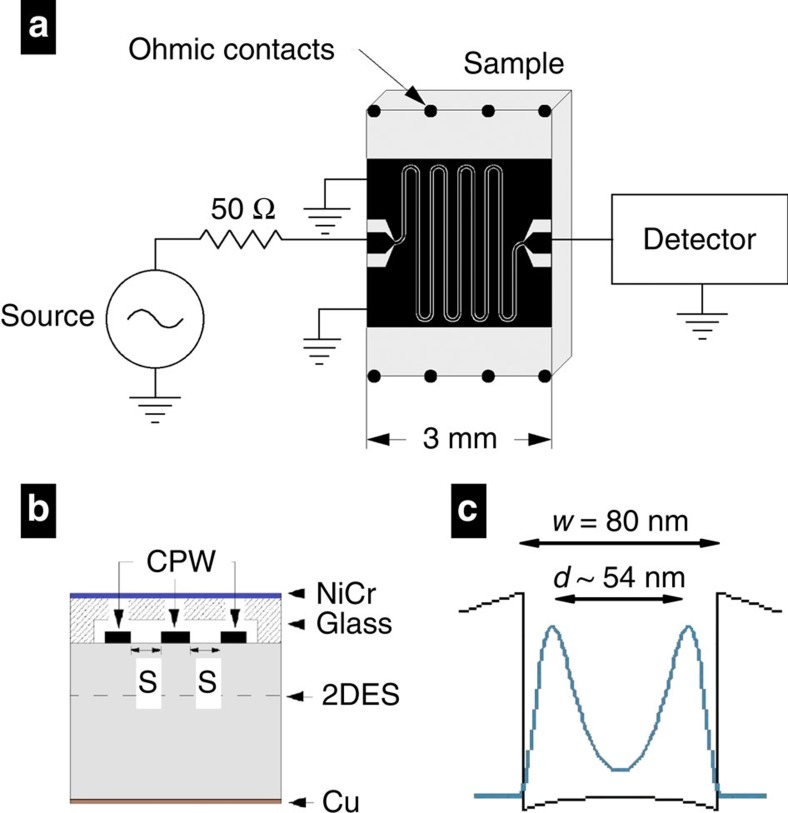
Schematic of sample for microwave measurement. (**a**) Schematic of the microwave measurement set-up. The source and detector are outside the cryostat at room temperature and the coplanar waveguide transmission line is patterned in metal film on top of the sample surface. The microwave conductivity *σ*_*xx*_ is calculated from loss through the line. (**b**) The microwave set-up shown in a cutaway side view. Slots of width *s* separate the centre, driven conductor and the ground planes. (**c**) The growth-direction electron charge distribution for a well of width *w*=80 nm at *n*=1.26 × 10^11^ cm^−2^ obtained from one-dimensional simulations. The growth-direction separation of charge density peaks is ∼54 nm.

**Figure 3:**
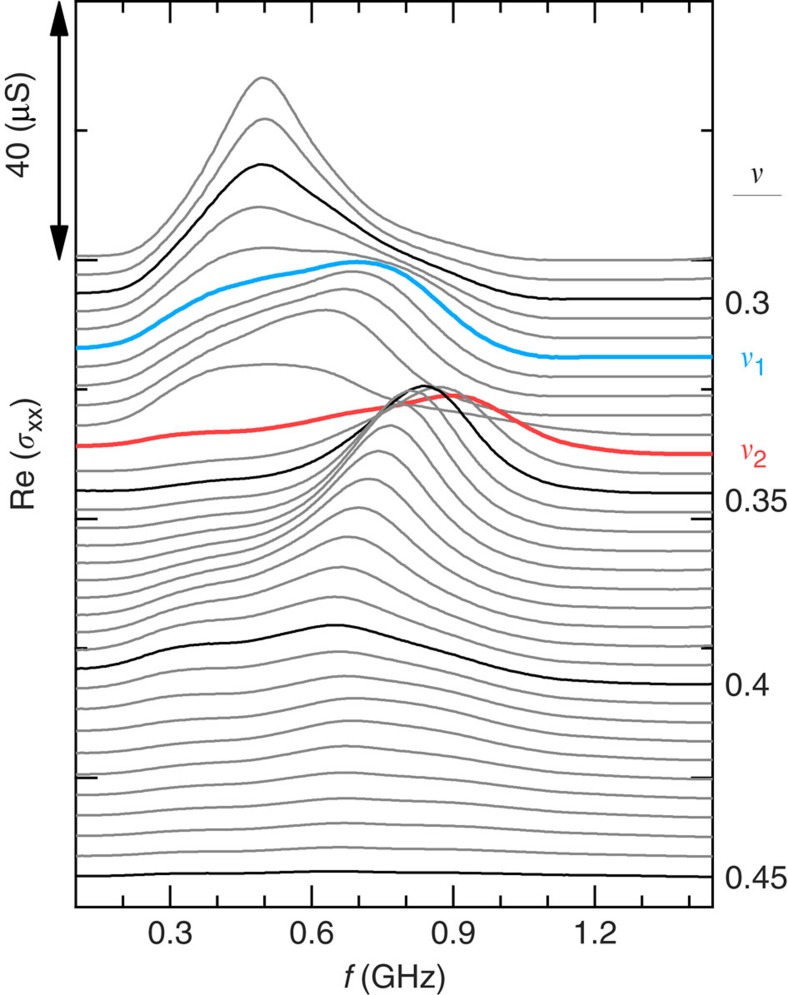
Two transitions in microwave spectra. Microwave spectra, Re (*σ*_*xx*_), versus frequency, *f*, at fixed *ν* from 0.29 to 0.45 vertically offset for clarity as marked on the right axis with step of 0.005, at *n*=1.26. Vertical scale is marked on the left axis. The spectra at low *ν* are dominated by a pinning mode resonance attributed to an electron solid. The spectra suggest the occurrence of two transitions within the solid, one at *ν*=0.315 and the other at *ν*=0.34, marked as *ν*_1_ and *ν*_2_, respectively. At each transition the peak frequency, *f*_pk_, exhibits an abrupt upward jump, and the amplitude of the resonance exhibits a minimum. The broad, weak peak at ∼0.3 GHz is an artefact because of a reflection near the sample mounting.

**Figure 4:**
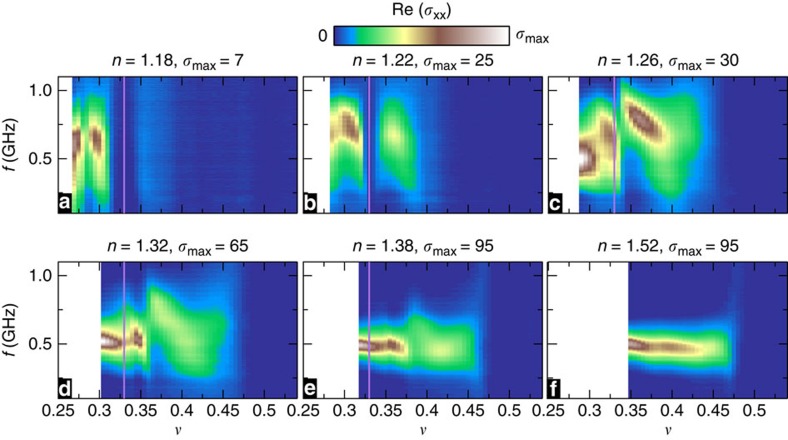
Electron density dependence of the transition. Plots of the microwave spectra, Re (*σ*_*xx*_), in the (*ν*, *f*) plane at various densities, *n*. *σ*_max_ is the largest Re (*σ*_*xx*_) on the colour scale. Vertical lines mark *ν*=1/3 and the solid white area at low *ν* is outside the magnetic field measurement range. (**a**) For *n*=1.18, the FQHE around *ν*=1/3 appears as a region with a flat spectrum and vanishing Re (*σ*_*xx*_)=0. A resonance is seen only for *ν*≤0.31, just below the FQHE state. At *ν*=0.285, the resonance peak (*σ*_pk_) in Re (*σ*_*xx*_) has a sharp minimum, interpreted as a transition within the solid. Possibly coincidentally, this *ν* is within error of 2/7. (**b**) On increasing to *n*=1.22, the resonance is re-entrant around the 1/3 FQHE and a minimum in *σ*_pk_ is located at *ν*=0.300. (**c**) For *n*=1.26, a resonance has replaced the FQHE at *ν*=1/3, while for *ν*=0.315 and 0.340 there are *σ*_pk_ minima, which are accompanied by jumps in *f*_pk_. (**d**,**e**) Data for *n*=1.32 and *n*=1.38, respectively. Each plot shows two minima in *σ*_pk_ with jumps in *f*_pk_. The jump in *f*_pk_ is larger for the higher-*ν* (*ν*_2_) minimum in *σ*_pk_. For *ν* above *ν*_2_ the resonance is broader with lower *σ*_pk_. The jump in *f*_pk_ for the lower-*ν* (*ν*_1_) transition at *n*=1.38 is much weaker than it is for lower *n*. (**f**) Data for *n*=1.52, at which both transitions have disappeared, and *f*_pk_ and the resonance amplitude vary only marginally throughout the measurement range.

**Figure 5:**
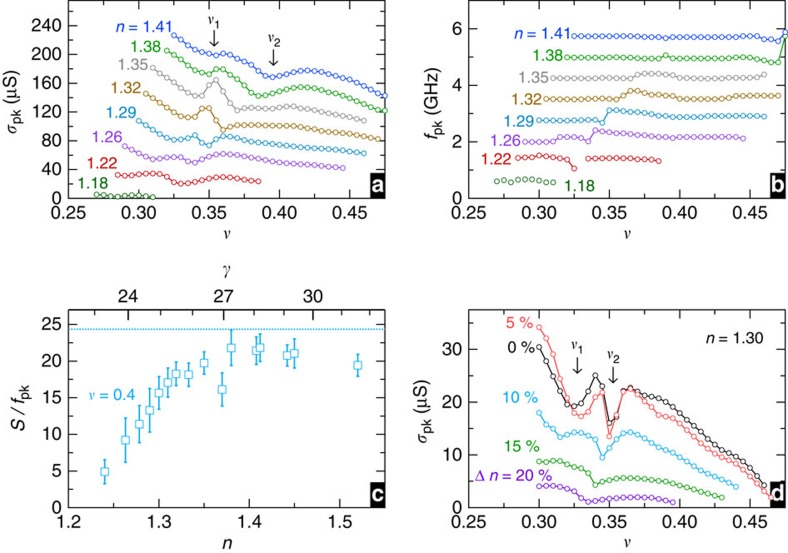
Electron density and Landau filling dependence of resonance parameters. (**a**) The resonance peak conductivity, *σ*_pk_, versus *ν* for many *n* successively offset by 20 μS. (**b**) The peak frequency *f*_pk_ versus *ν* for the same *n* as (**a**) successively offset by 0.5 GHz. Spectra were taken at *ν* intervals of 0.005. We denote the lower and higher *ν* minima, respectively, by *ν*_1_ and *ν*_2_. (**c**) *S*/*f*_pk_ versus *n*, where *S* is the integrated Re (*σ*_*xx*_) versus *f* for the resonance, for *ν*=0.4 (where the integration is performed for 0.1<*f*<1.5 GHz). Conversion to *γ* is plotted on the top axis. The error in *S*/*f*_pk_ is calculated as the difference between the measured value and that obtained by fitting the resonance to a Gaussian. The dotted line marks the theoretical value^15^ *S*/*f*_pk_=*πe*^2^*ν*/2*h* for full-charge carrier participation. Except near *n*=1.38, at which *S*/*f*_pk_ is suppressed, the curve increases before saturating at ∼85% of the theoretical value. The observed increase in *S*/*f*_pk_ demonstrates increase in the strength of the bilayer solid with increasing *n* as the available particles contribute to the solid. (**d**) The peak resonance *σ*_pk_ versus *ν* at fixed *n*=1.30. Here the well is imbalanced by asymmetric gating to transfer charge Δ*n* between the two layers.

**Figure 6:**
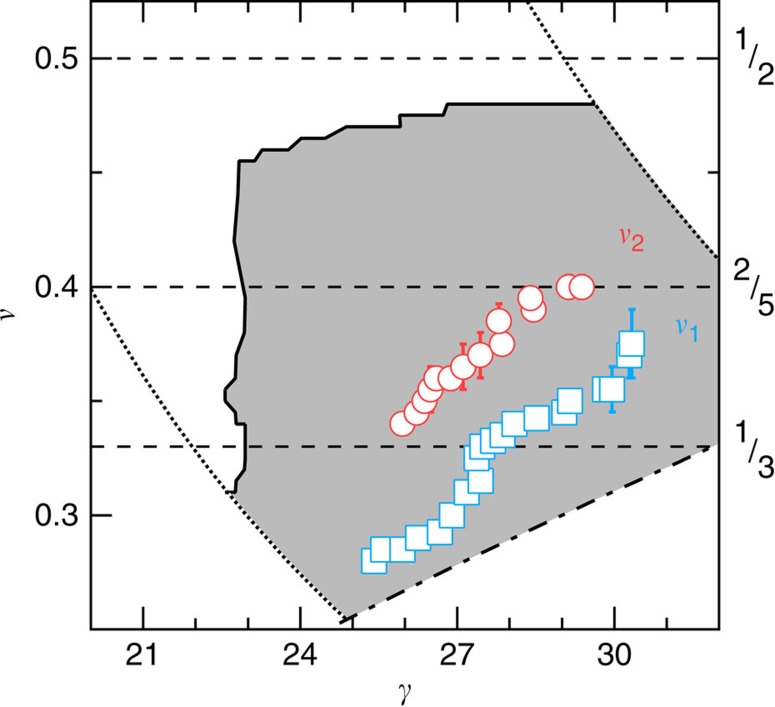
Phase diagram in the *γ*–*ν* plane. The shaded region is where microwave spectra show a resonance of amplitude *σ*_pk_>2 μS. The edges of the measurement range resulting from limits in the sample density range obtainable from gating are shown as dotted lines. The measurement range limit due to the maximum magnetic field is the upward sloping dashed-dotted line. The squares and circles denote the transition filling factors, *ν*_1_ and *ν*_2_, respectively, as identified from minima in the resonance peak conductivity, *σ*_pk_, versus *ν* with error due to the width of the minimum.

Our microwave spectroscopy technique^18^,^22^ uses a coplanar waveguide (CPW) on the surface of a sample. A NiCr front gate was deposited on glass that was etched to space it from the CPW by ∼10 μm. A schematic diagram of the microwave measurement technique is shown in Fig. 2a, and a cutaway view of the sample is shown in Fig. 2b. In the high-frequency, low-loss limit, diagonal conductivity is approximated by *σ*_*xx*_(*f*)=(*s*/*lZ*_0_)ln(*t*/*t*_0_), where *s*=30 μm is the distance between the centre conductor and ground plane, *l*=28 mm is the length of the CPW and *Z*_0_=50 Ω is the characteristic impedance without the 2DES. *t* is the amplitude at the receiver and *t*_0_ is the normalizing amplitude. The normalizing amplitude was taken at *ν*=1/2; the difference on using *ν*=1 rather than 1/2 as a reference is less than 1 μS. Hence, *σ*_*xx*_(*f*) is the difference between the conductivity and that for *ν*=0.5. The microwave measurements were carried out in the low-power limit, in which the measurement is not sensitive to the excitation power.

### Field modelling

We calculate the reported Re (*σ*_xx_) data from *t* using the results of modelling the fields and currents of the system of the CPW coupled to the WQW, without requiring a high-*f* low-loss limit. As in ref. 21, the model calculation is based on ref. 32. Let *x* be the distance from the CPW centre, measured perpendicular to the propagation direction. *φ*_0_(*x*) is the potential with 1 V on the centre conductor and 0 V on the ground plane. 
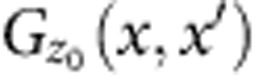
 is the electrostatic potential Green's function. 
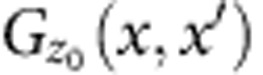
 and *φ*_0_(*x*) are found by conformal mapping taking *σ*_*xx*_≡0. Assuming *σ*_*xx*_ to be wave vector-independent, the quasistatic potential, *φ*(*x*), is found from the numerical solution of


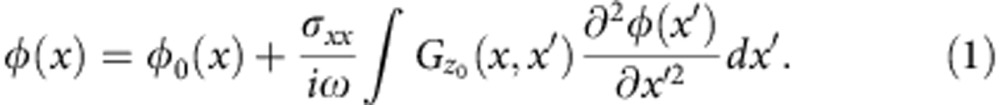


*φ*(*x*) is then differentiated to obtain the nonequilibrium charge, from which we calculate the induced charge on the CPW. We then calculate the per-unit-length admittance *Y*_L_, the *σ*_*xx*_-independent per-unit-length CPW series impedance *Z*_L_, and calculate the complex transmission coefficient *s*_21_ of the line using lossy transmission-line models^32^. Finally, the measured *σ*_*xx*_ is found by iteratively comparing measured *t*/*t*_0_ to the calculated *s*_21_(*σ*_*xx*_, *f*)/*s*_21_(0, *f*). The result is a correction of at most a factor of two from the approximate formula. The correction had no effect on measured peak frequencies.

## Author contributions

A.T.H. conceived and designed the experiment, performed the microwave measurements, analysed the data and co-wrote the manuscript. Y.L. performed computer simulations, discussed data analysis and co-wrote the manuscript. L.W.E. conceived and designed the experiment, discussed data analysis and co-wrote the manuscript. M.S. conceived the experiment, discussed data analysis and co-wrote the manuscript. L.N.P., K.W.W. and K.W.B. were responsible for the growth of the samples.

## Additional information

**How to cite this article:** Hatke, A.T. *et al*. Microwave spectroscopy of the low-filling-factor bilayer electron solid in a wide quantum well. *Nat. Commun.* 6:7071 doi: 10.1038/ncomms8071 (2015).
